# Supplementary material for: Individual differences in motives for costly punishment
Source: Commun Psychol. 2026 Jan 12;4:15. doi: 10.1038/s44271-025-00372-w (PMC12852156; doi:10.1038/s44271-025-00372-w)
Supplement: Supplementary file 2 — Supplementary Information [file 44271_2025_372_MOESM2_ESM.pdf]

Supplementary Materials for "Individual differences in motives for costly punishment"

Scott Claessens[1,2], Quentin D Atkinson[2], and Nichola J Raihani[2,3*]

[1]School of Psychology, University of Kent, Canterbury, United Kingdom

[2]School of Psychology, University of Auckland, Auckland, New Zealand

[3]Department of Experimental Psychology, University College London, London, United Kingdom

*Correspondence concerning this article should be addressed to Nichola J Raihani, Department of Experimental Psychology, University College London, London, United Kingdom, Email: n.raihani@ucl.ac.uk

This file contains:

- Supplementary Figures 1–15
- Supplementary Tables 1–4

Supplementary Figures

Supplementary Figure 1: Sample characteristics in the United Kingdom. N = 1014 participants.

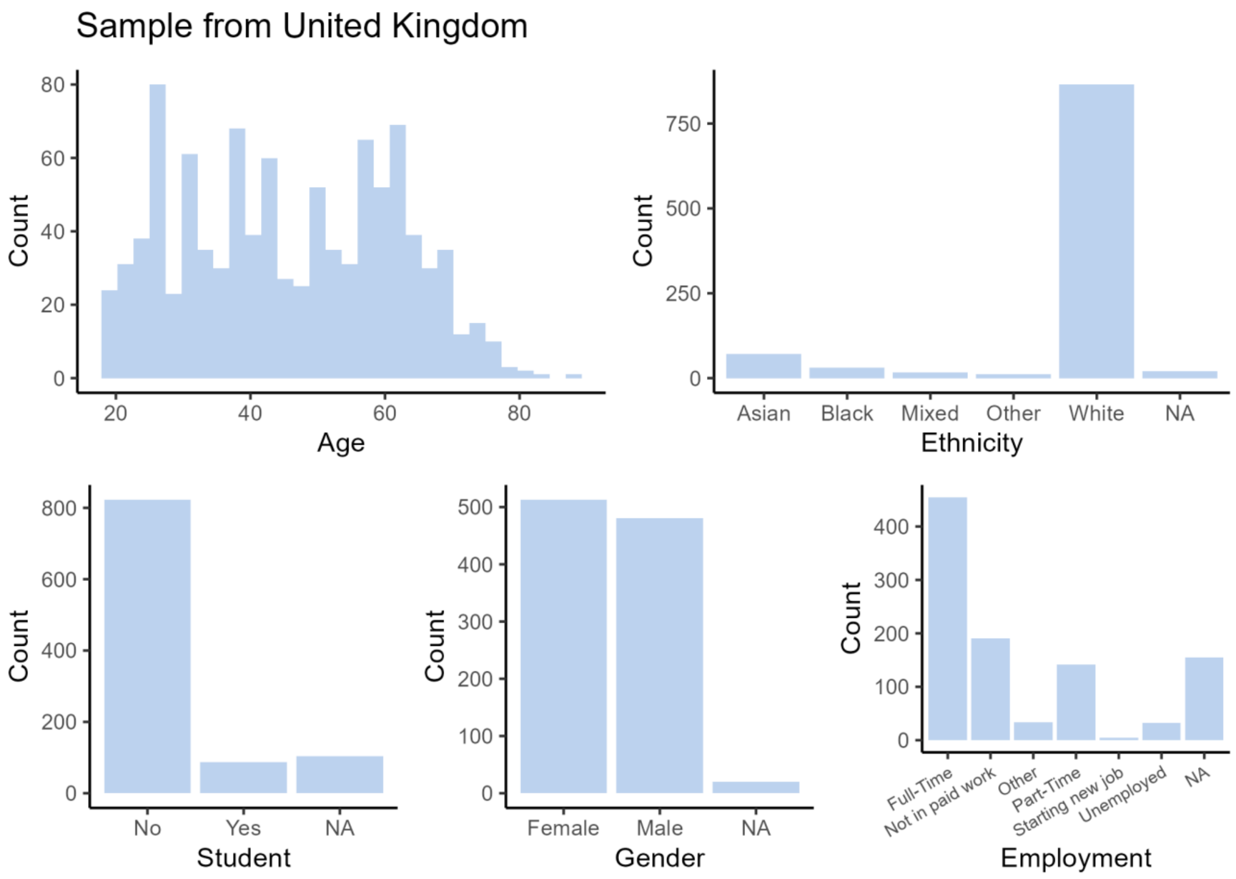

Supplementary Figure 2: Sample characteristics in the United States. N = 996 participants.

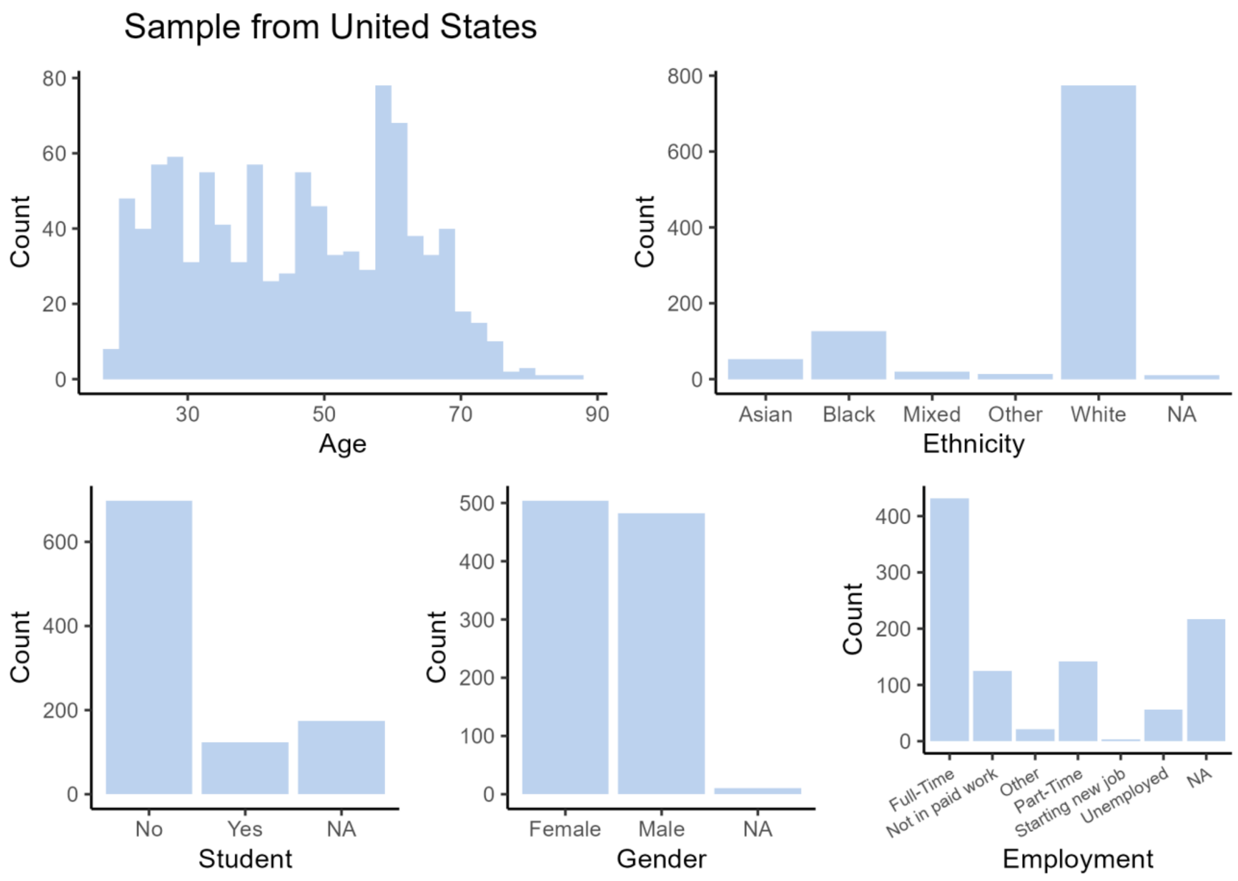

Supplementary Figure 3: Posterior estimates of the probabilities of following different punishment strategies from the Bayesian latent state model that estimates the implementation error rate as a free parameter. The model estimated the implementation error rate to be 0.03 (95% CI [0.00 0.06]). Points represent posterior medians, line ranges represent 50% and 95% credible intervals. N = 2010 participants. AI = advantageous inequity, DI = disadvantageous inequity.

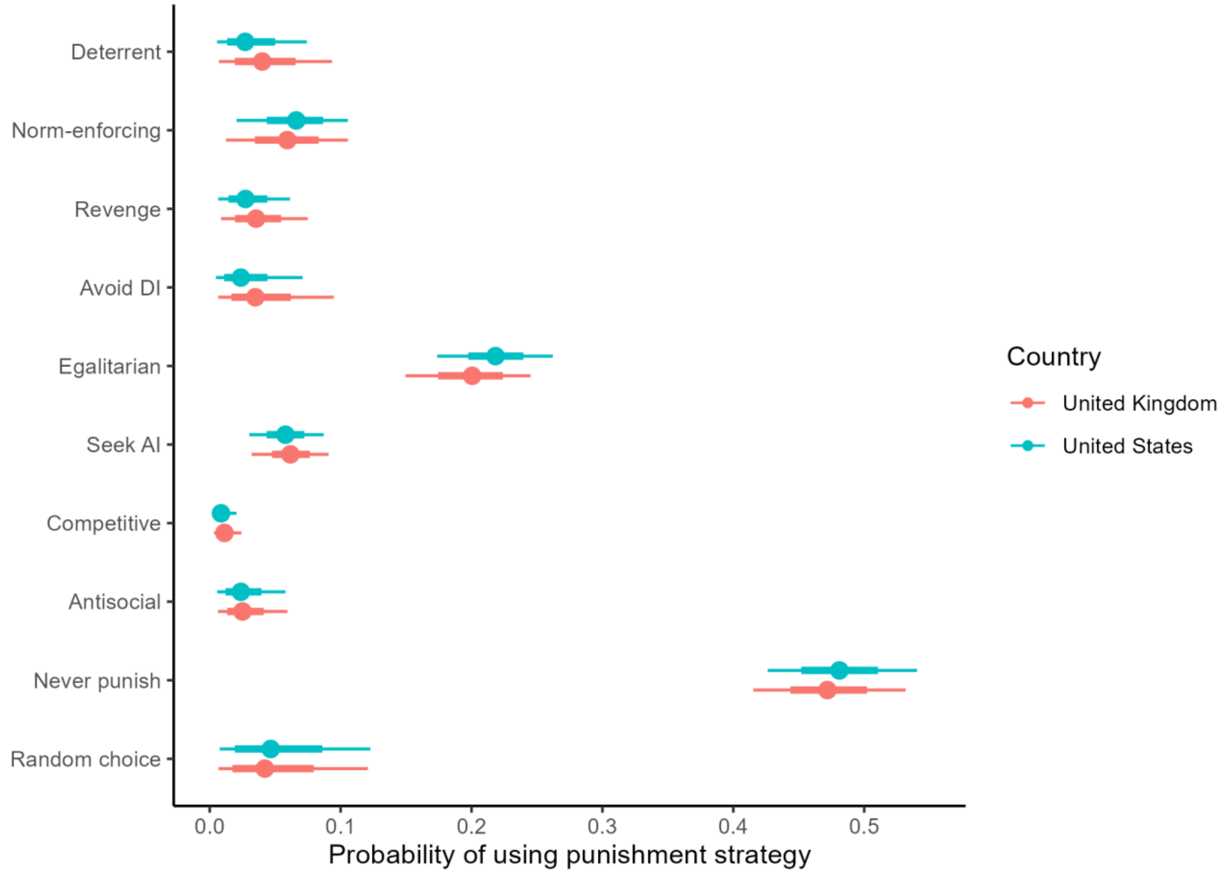

Supplementary Figure 4: MCMC trace plots for parameter values from the Bayesian latent state model fitted to data with exclusions. These trace plots suggest that the different chains mixed well and the model converged normally.

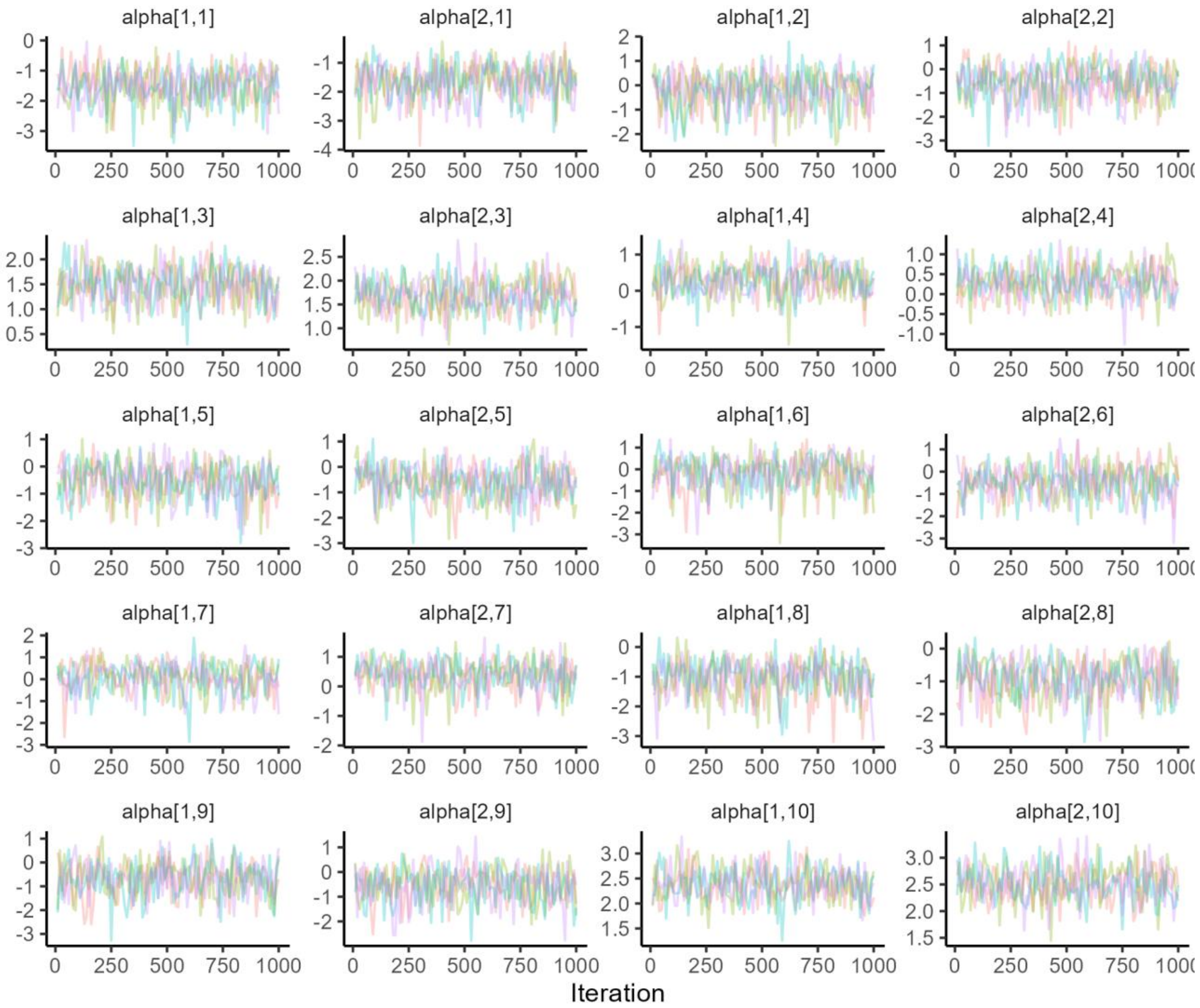

Supplementary Figure 5: Results of Bayesian latent state model fitted to simulated data (n = 100) with known strategy frequencies in the population. Blue points represent known strategy frequencies, grey densities represent posterior estimates of strategy frequencies. AI = advantageous inequity, DI = disadvantageous inequity.

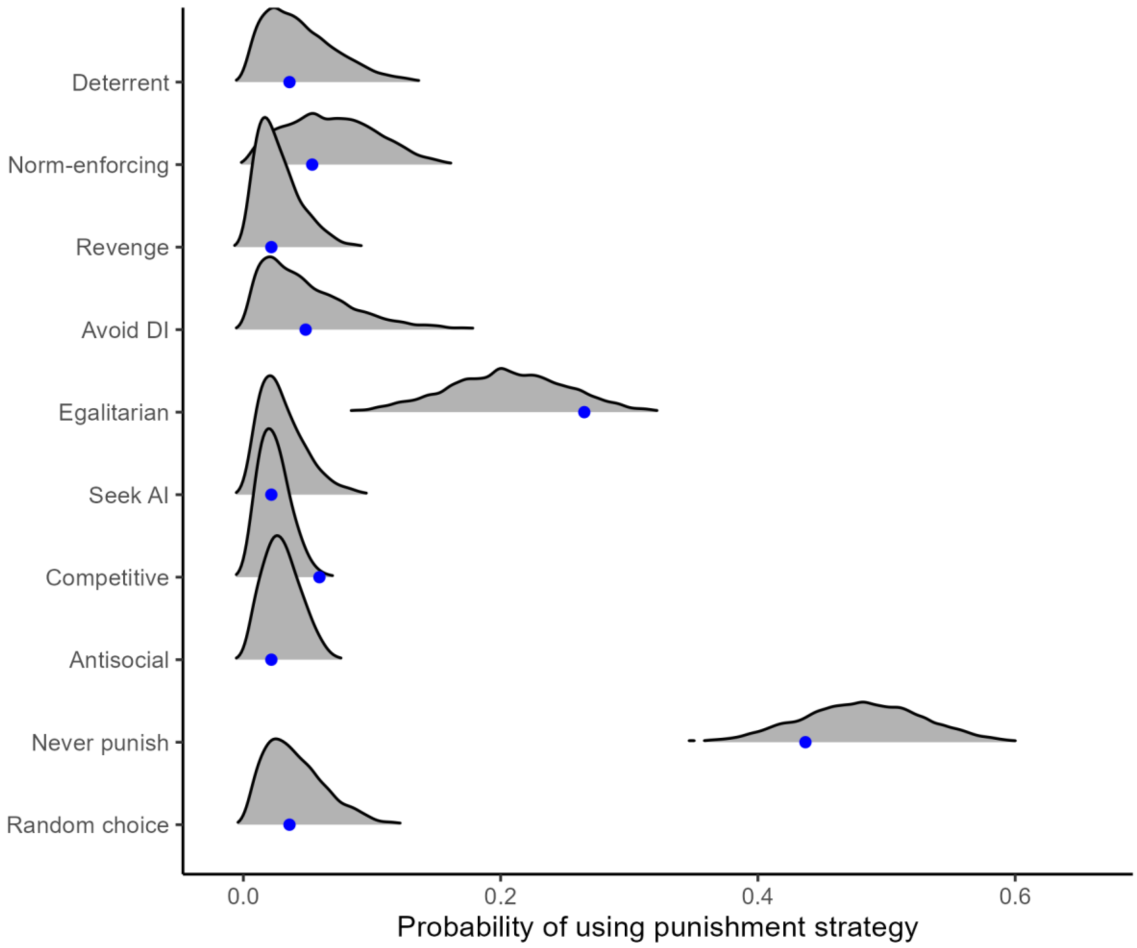

Supplementary Figure 6: Posterior estimates of the probabilities of following different punishment strategies from the Bayesian latent state model fitted to the full dataset without pre-registered exclusions. The model assumes an implementation error rate of 5%. Figure 3 in the main text shows the same result, but from a model fitted to the reduced dataset with pre-registered exclusions. Points represent posterior medians, line ranges represent 50% and 95% credible intervals. N = 2024 participants. AI = advantageous inequity, DI = disadvantageous inequity.

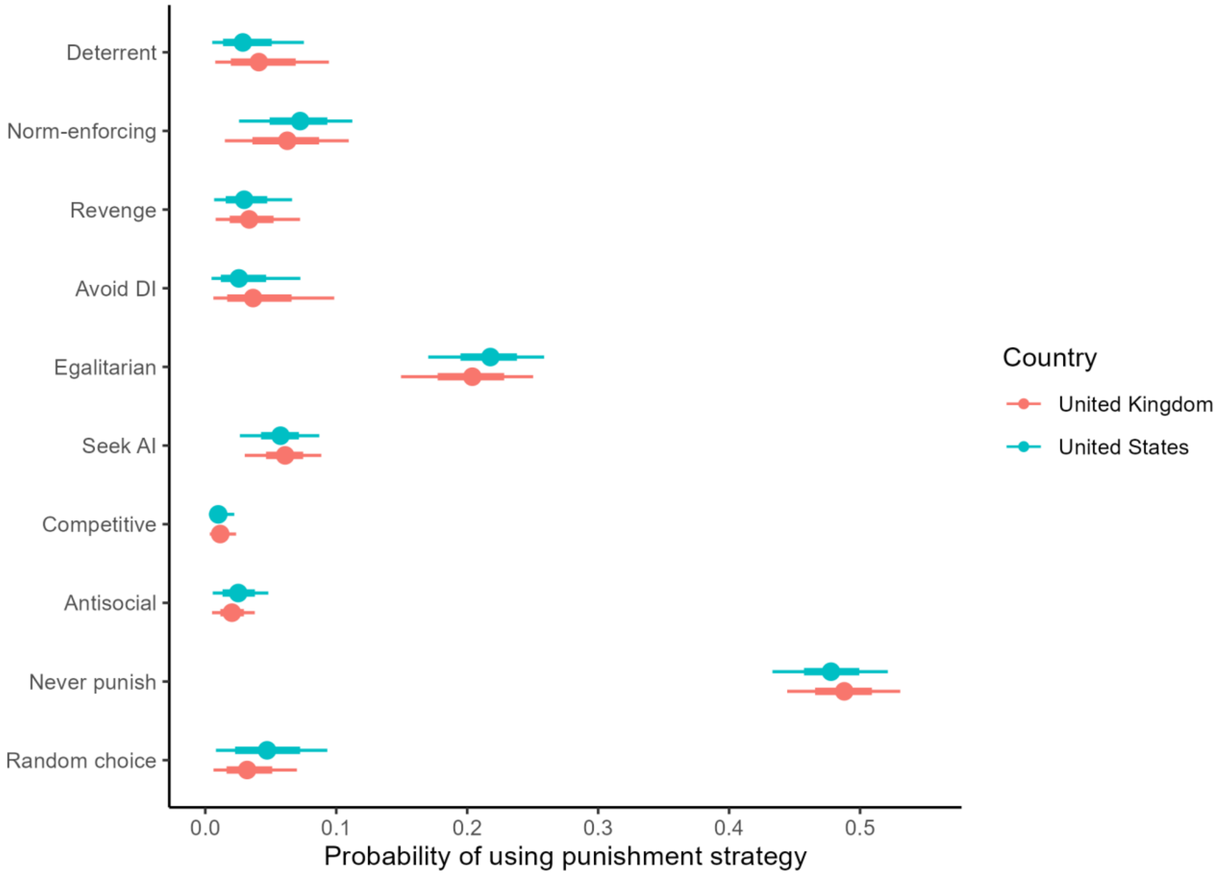

Supplementary Figure 7: Posterior estimates of the probabilities of following different punishment strategies from the Bayesian latent state model include a "third-party" strategy that punishes only when the target steals in the third-party game (Game F). The model was fitted to the full dataset with pre-registered exclusions. The model assumes an implementation error rate of 5%. Points represent posterior medians, line ranges represent 50% and 95% credible intervals. N = 2010 participants. AI = advantageous inequity, DI = disadvantageous inequity.

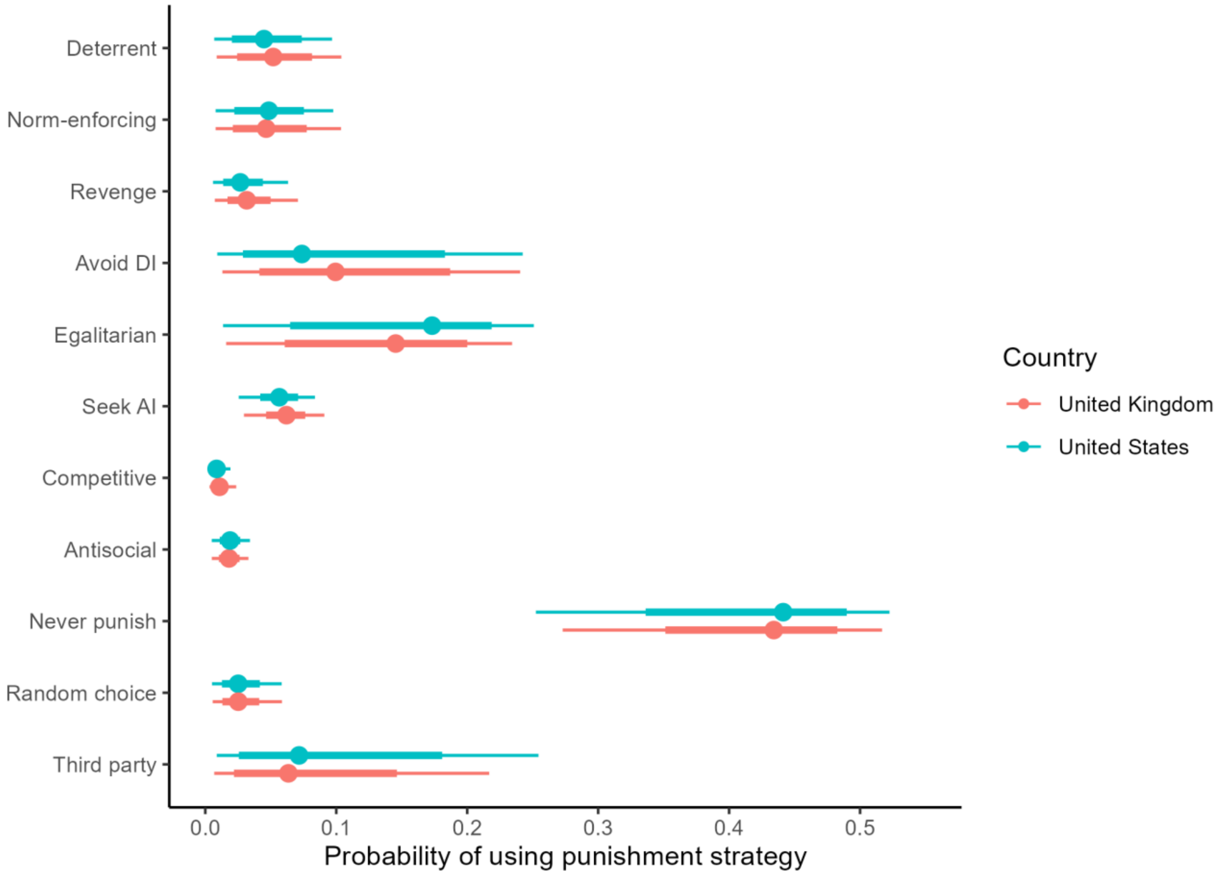

Supplementary Figure 8: Posterior slopes from models including age, socio-economic status, gender, and student status, fitted to the subsetted dataset with pre-registered exclusions. Each row represents a separate model. Points represent posterior medians, line ranges represent 95% credible intervals. N = 2010 participants. AI = advantageous inequity, DI = disadvantageous inequity.

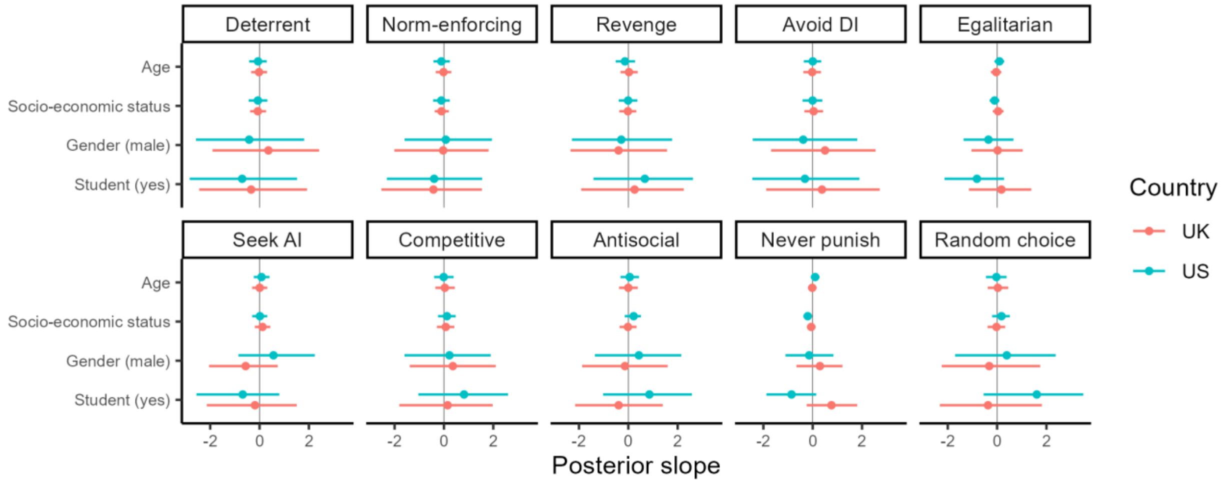

Supplementary Figure 9: Posterior slopes from models including age, socio-economic status, gender, and student status, fitted to the full dataset without pre-registered exclusions. Each row represents a separate model. Points represent posterior medians, line ranges represent 95% credible intervals. N = 2024 participants. AI = advantageous inequity, DI = disadvantageous inequity.

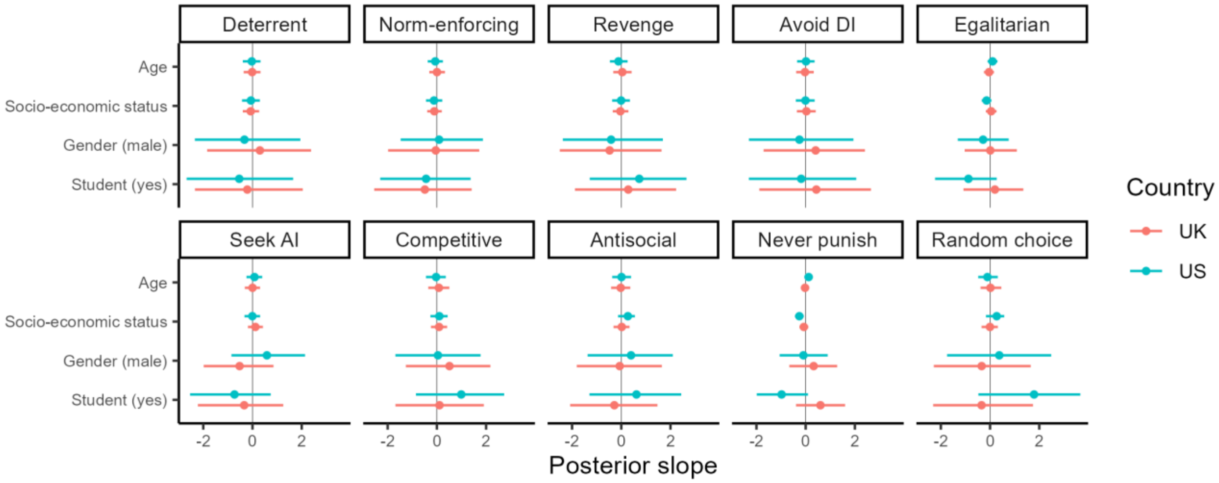

Supplementary Figure 10: Posterior slopes from models including Big-6 personality dimensions and Social Value Orientation, fitted to the full dataset without pre-registered exclusions. Each row represents a separate model. Figure 4 in the main text shows the same results, but from models fitted to the reduced dataset with pre-registered exclusions. Points represent posterior medians, line ranges represent 95% credible intervals. N = 2024 participants. AI = advantageous inequity, DI = disadvantageous inequity.

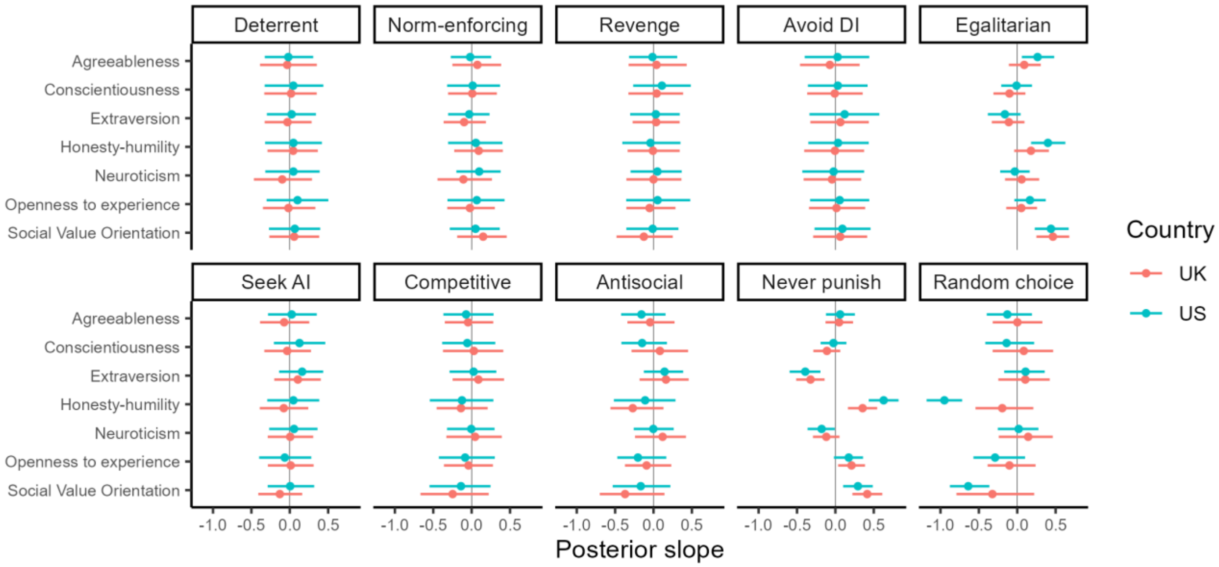

Supplementary Figure 11: Posterior slopes from models including political ideology, views about social inequality, and religiosity, fitted to the full dataset without pre-registered exclusions. Each row represents a separate model aside from Social Dominance Orientation and Right Wing Authoritarianism, which control for one another within the same model. Figure 5 *in the main text shows the same results, but from models fitted to the reduced dataset with pre-registered exclusions. Points represent posterior medians, line ranges represent 95% credible intervals. N = 2024 participants. AI = advantageous inequity, DI = disadvantageous inequity.*

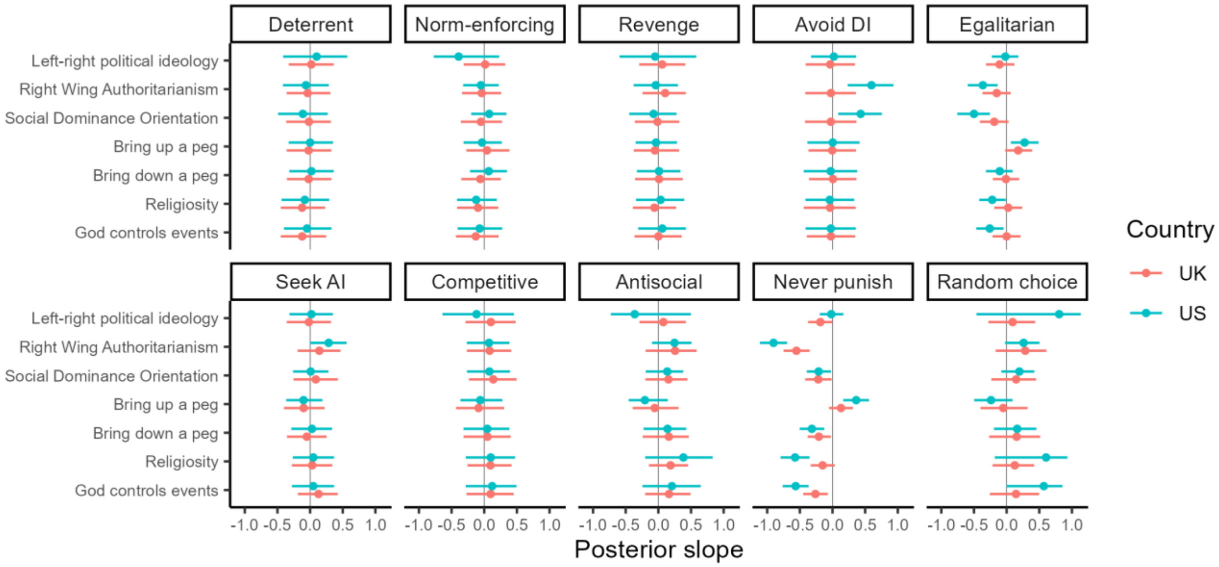

Supplementary Figure 12: Boxplots showing the distribution of responses to each self-report question about the reasons for participants' behaviour in the games, presented as deviations from participants' average rating across all questions. Boxplots represent medians and interquartile ranges. N = 2010 participants.

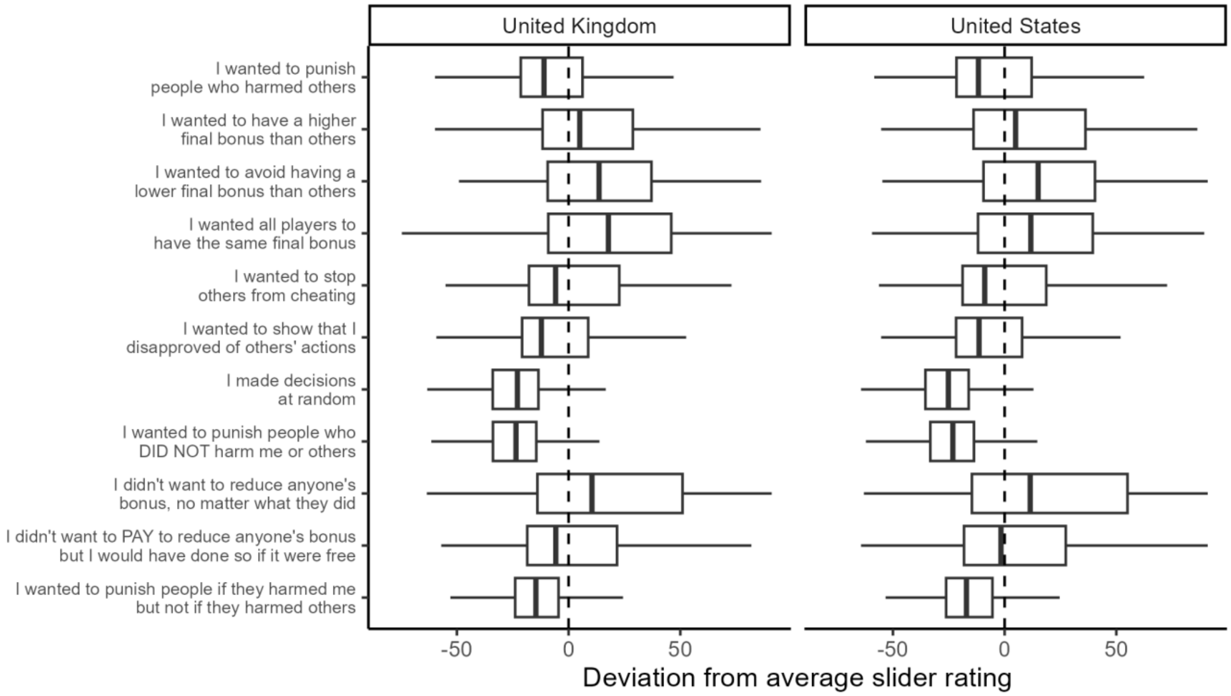

Supplementary Figure 13: Posterior slopes from models including self-reported strategy usage, fitted to the full dataset without pre-registered exclusions. Each row represents a separate model. Highlighted estimates represent combinations where the self-report slider matched the behavioural strategy. Figure 7 in the main text shows the same results, but from models fitted to the reduced dataset with pre-registered exclusions. Points represent posterior medians, line ranges represent 95% credible intervals. N = 2024 participants. AI = advantageous inequity, DI = disadvantageous inequity.

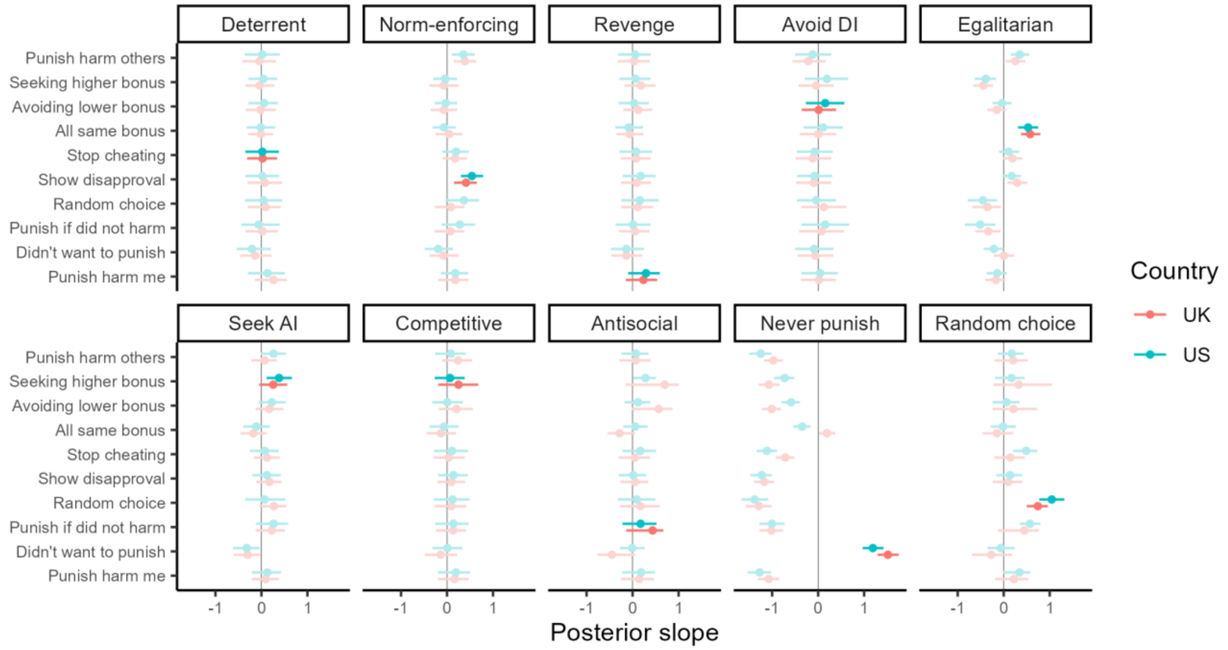

Supplementary Figure 14: Correlation matrix visualising the Spearman's rank correlations between the self-reported intentions slider questions. N = 2010 participants.

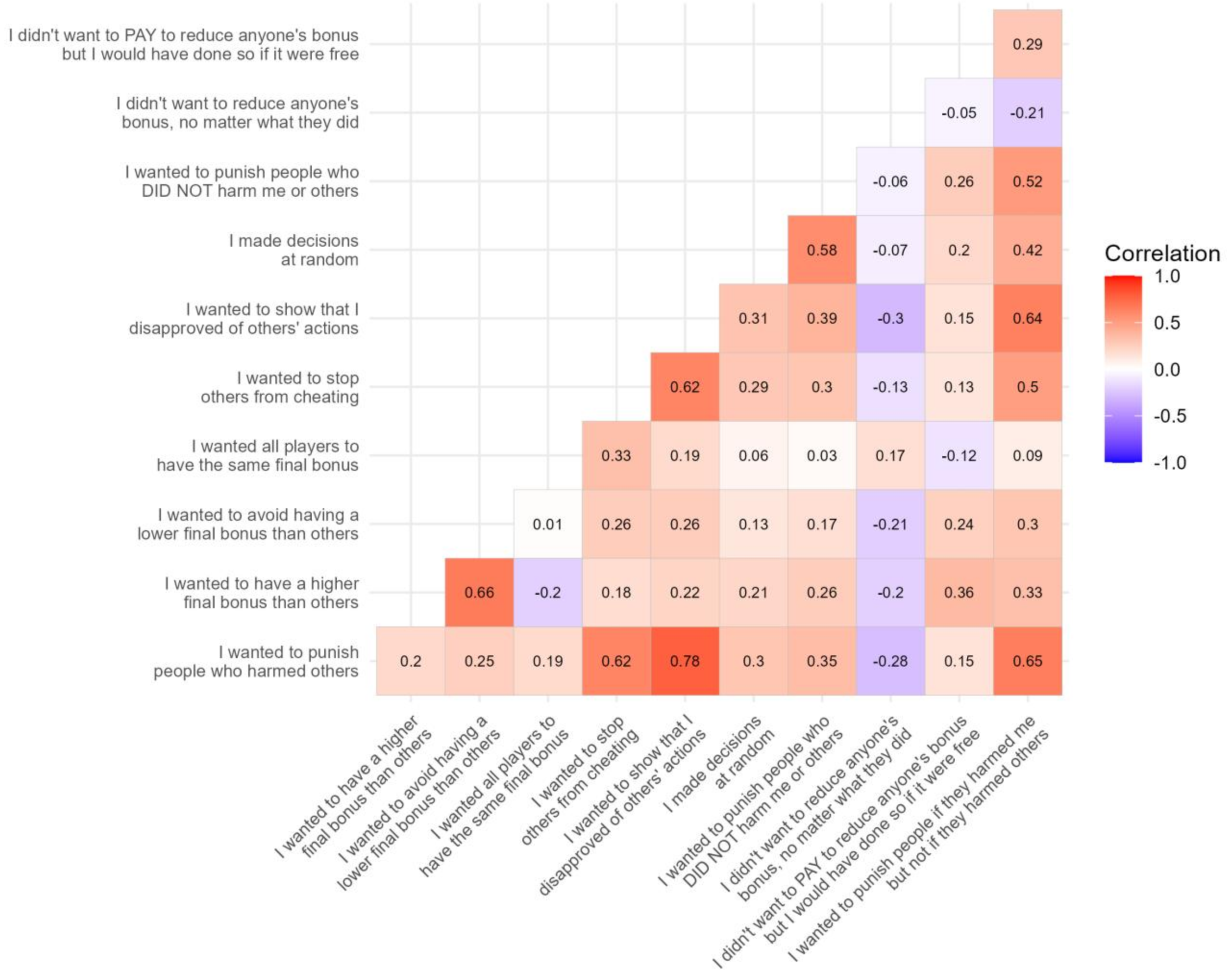

Supplementary Figure 15: The relationships between strategy frequencies and the overall costs of strategies across all twelve punishment decisions, in both countries. Each point is a unique strategy that appears in our dataset at least once (for ease of presentation, the "never punish" strategy is excluded). Lines and shaded areas represent posterior predictions from splines fitted to the trend in each country separately. N = 2024 participants.

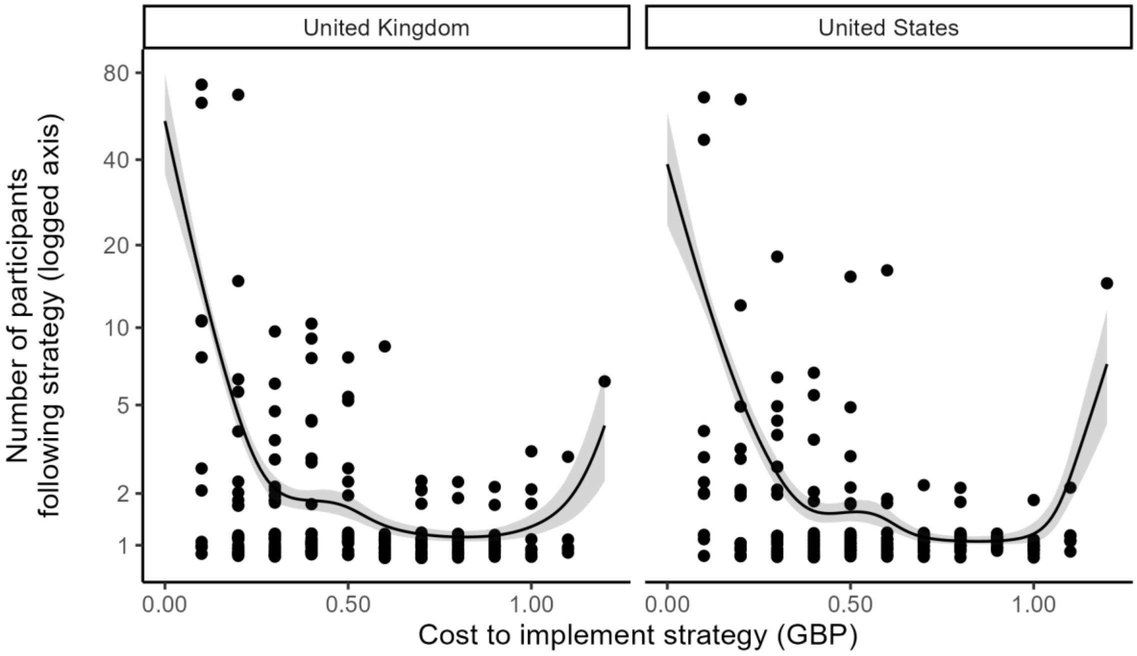

Supplementary Tables

Slider	Wording
1	I wanted to punish people who harmed others
2	I wanted to have a higher final bonus than others
3	I wanted to avoid having a lower final bonus than others
4	I wanted all players to have the same final bonus
5	I wanted to stop others from cheating
6	I wanted to show that I disapproved of others' actions
7	I made decisions at random
8	I wanted to punish people who DID NOT harm me or others
9	I didn't want to reduce anyone's bonus, no matter what they did
10	I didn't want to PAY to reduce anyone's bonus but I would have done so if it were free
11	I wanted to punish people if they harmed me but not if they harmed others

Supplementary Table 1: Wordings for 11 self-report slider questions asking participants to report the reasons for their behaviour in the six games. Participants were prompted with the following text: "We would now like you to answer a few questions about your main motivation in the games. Please answer truthfully - there is no right or wrong answer and your first answer is probably best. Please rate the extent to which the following statements apply to your decisions to reduce or not to reduce other players' bonuses in the games."

Measure	Wording	Scale
Demographics	What is your highest level of education?	
	Where would you place yourself on this ladder? Please indicate which number on the rung best represents where you stand at this time in your life, relative to other people in your country	
	Please could you tell us roughly how many years have you lived in your current country of residence?	
Big 6 Extraversion	I am the life of the party	1-7
	I don't talk a lot (reversed)	1-7
	I keep in the background (reversed)	1-7
	I talk to a lot of different people at parties	1-7
Big 6 Agreeableness	I sympathise with others' feelings	1-7
	I am not interested in other people's problems (reversed)	1-7
	I feel others' emotions	1-7
	I am not really interested in others (reversed)	1-7
Big 6 Conscientiousness	I get chores done right away	1-7
	I like order	1-7
	I make a mess of things (reversed)	1-7
	I often forget to put things back in their proper place (reversed)	1-7
Big 6 Neuroticism	I have frequent mood swings	1-7
	I am relaxed most of the time (reversed)	1-7
	I get upset easily	1-7
	I seldom feel blue (reversed)	1-7
Big 6 Openness to experience	I have a vivid imagination	1-7
	I have difficulty understanding abstract ideas	1-7
	I do not have a good imagination (reversed)	1-7
	I am not interested in abstract ideas (reversed)	1-7

Big 6 Honesty-humility	I feel entitled to more of everything (reversed)	1-7
	I deserve more things in life (reversed)	1-7
	I would like to be seen driving around in a very expensive car (reversed)	1-7
	I would get a lot of pleasure from owning expensive luxury goods (reversed)	1-7
Social Value Orientation	Please indicate how you would like to distribute money between yourself and the other player	9 choices
Left-right political ideology	Political views are often organised on a single scale from left to right. For example, in the United States, the Democratic Party is described as more to the left and the Republican Party is described as more to the right. If you had to place your political views on this left-right scale, generally speaking, where would you put yourself?	0-100 slider
Social Dominance Orientation	An ideal society requires some groups to be on top and others to be on the bottom	1-7
	Some groups of people are simply inferior to other groups	1-7
	No one group should dominate in society (reversed)	1-7
	Groups at the bottom are just as deserving as groups at the top (reversed)	1-7
	Group equality should not be our primary goal	1-7
	It is unjust to try to make groups equal	1-7
	We should do what we can to equalize conditions for different groups (reversed)	1-7
	We should work to give all groups an equal chance to succeed (reversed)	1-7
Right Wing Authoritarianism	It's great that many young people today are prepared to defy authority (reversed)	1-9
	What our country needs most is discipline, with everyone following our leaders in unity	1-9
	God's laws about abortion, pornography, and marriage must be strictly followed before it is too late	1-9
	There is nothing wrong with premarital sexual intercourse (reversed)	1-9

	Our society does NOT need tougher government and stricter laws (reversed)	1-9
	The facts on crime and the recent public disorders show we have to crack down harder on troublemakers, if we are going to preserve law and order	1-9
Views on social inequality	I would like to bring the people above me on the ladder down a peg or two	1-7
	I would like to bring the people below me on the ladder up a peg or two	1-7
Religious views	How religious are you?	1-5
	It is likely that God, or some other type of spiritual non-human entity, controls the events in the world	1-7

Supplementary Table 2: Wordings for survey questions in the study.

Game	United Kingdom	United States
Game A (AI)	0.96	0.94
Game B (Equal)	0.95	0.93
Game C (Computer)	0.95	0.95
Game D (1:1 Fee-Fine)	0.95	0.94
Game E (DI)	0.96	0.94
Game F (Third-Party)	0.95	0.94

Supplementary Table 3: Proportions of correct answers to comprehension questions for all six economic games, split by country.

		United Kingdom (N = 1014)		United States (N = 996)	
Pattern	Explanation	N	Prop	N	Prop
000000000000	Never punish strategy (exact)	426	0.420	447	0.449
000000001000	Avoid DI strategy (exact)	67	0.066	62	0.062
000000001010	Egalitarian strategy (exact)	65	0.064	71	0.071
000000000010	Punish when steal in Game F	55	0.054	49	0.049
001000001000	Punish when steal in Games B and E	14	0.014	11	0.011
101000001010	Punish when steal in Games A, B, E, and F	11	0.011	4	0.004
100000000000	Punish when steal in Game A	10	0.010	2	0.002
000000100000	Punish when steal in Game D	9	0.009	3	0.003
001000001010	Punish when steal in Games B, E, and F	9	0.009	17	0.017
101000101000	Deterrent strategy (exact)	9	0.009	6	0.006
101010101010	Punish when steal in all games	9	0.009	15	0.015
101000101010	Norm-enforcing strategy (exact)	8	0.008	16	0.016
001000000000	Punish when steal in Game B	7	0.007	4	0.004
001010101000	Punish when steal in Games B, C, D, and E	7	0.007	0	0.000
100000001000	Punish when steal in Games A and E	6	0.006	5	0.005
101000001000	Punish when steal in Games A, B, and E	6	0.006	7	0.007
101010101000	Revenge strategy (exact)	6	0.006	5	0.005
111111111111	Always punish	6	0.006	16	0.016
000000101000	Punish when steal in Games D and E	5	0.005	2	0.002
000000101010	Punish when steal in Games D, E, and F	5	0.005	3	0.003
101010001010	Punish when steal in all games except Game D	5	0.005	2	0.002
001000101000	Punish when steal in Games B, D, and E	4	0.004	2	0.002
001000101010	Punish when steal in Games B, D, E, and F	4	0.004	6	0.006
101000000000	Punish when steal in Games A and B	4	0.004	2	0.002
101010001000	Punish when steal in Games A, B, C, and E	4	0.004	0	0.000

Supplementary Table 4: Counts and proportions of the 25 most common patterns of punitive behaviour across all twelve decisions, split by country. Binary strings represent punishment (1) or no punishment (0) in each decision, aligning with the order of game decision columns in Table 1.
