# Supplementary material for: Individual differences in motives for costly punishment
Source: Commun Psychol. 2026 Jan 12;4:15. doi: 10.1038/s44271-025-00372-w (PMC12852156; doi:10.1038/s44271-025-00372-w)
Supplement: Supplementary file 3 — Reporting Summary [file 44271_2025_372_MOESM3_ESM.pdf]

nature portfolio

Corresponding author(s): Scott Claessens

Last updated by author(s): Oct 8, 2025

Reporting Summary

Nature Portfolio wishes to improve the reproducibility of the work that we publish. This form provides structure for consistency and transparency in reporting. For further information on Nature Portfolio policies, see our Editorial Policies and the Editorial Policy Checklist.

Statistics

For all statistical analyses, confirm that the following items are present in the figure legend, table legend, main text, or Methods section.

n/a	Confirmed	
☐	☒	The exact sample size (*n*) for each experimental group/condition, given as a discrete number and unit of measurement
☐	☒	A statement on whether measurements were taken from distinct samples or whether the same sample was measured repeatedly
☐	☒	The statistical test(s) used AND whether they are one- or two-sided<br>*Only common tests should be described solely by name; describe more complex techniques in the Methods section.*
☐	☒	A description of all covariates tested
☐	☒	A description of any assumptions or corrections, such as tests of normality and adjustment for multiple comparisons
☐	☒	A full description of the statistical parameters including central tendency (e.g. means) or other basic estimates (e.g. regression coefficient) AND variation (e.g. standard deviation) or associated estimates of uncertainty (e.g. confidence intervals)
☐	☒	For null hypothesis testing, the test statistic (e.g. *F*, *t*, *r*) with confidence intervals, effect sizes, degrees of freedom and *P* value noted<br>*Give P values as exact values whenever suitable.*
☐	☒	For Bayesian analysis, information on the choice of priors and Markov chain Monte Carlo settings
☐	☒	For hierarchical and complex designs, identification of the appropriate level for tests and full reporting of outcomes
☐	☒	Estimates of effect sizes (e.g. Cohen's *d*, Pearson's *r*), indicating how they were calculated

Our web collection on statistics for biologists contains articles on many of the points above.

Software and code

Policy information about availability of computer code

Data collection	We did not use custom code to collect data in this study.
Data analysis	Code to reproduce the analyses, figures, and manuscript can be found here: https://github.com/ScottClaessens/punishStrategies

For manuscripts utilizing custom algorithms or software that are central to the research but not yet described in published literature, software must be made available to editors and reviewers. We strongly encourage code deposition in a community repository (e.g. GitHub). See the Nature Portfolio guidelines for submitting code & software for further information.

Data

Policy information about availability of data

All manuscripts must include a data availability statement. This statement should provide the following information, where applicable:

- Accession codes, unique identifiers, or web links for publicly available datasets
- A description of any restrictions on data availability
- For clinical datasets or third party data, please ensure that the statement adheres to our policy

All data can be accessed here: https://github.com/ScottClaessens/punishStrategies

Research involving human participants, their data, or biological material

Policy information about studies with human participants or human data. See also policy information about sex, gender (identity/presentation), and sexual orientation and race, ethnicity and racism.

Reporting on sex and gender	We collected data on participants' gender identity from Prolific demographics. Since we did not ask about biological sex, we do not use this terminology in the paper. Supplementary Figures 11 and 12 display numbers of male and female participants. We report gender-based analyses in Supplementary Figures 3 and 4.
Reporting on race, ethnicity, or other socially relevant groupings	Data on ethnicity, nationality, and country of birth were collected from Prolific demographics. Supplementary Figures 11 and 12 display the sample breakdown by ethnicity. However, we do not refer to these socially-relevant groupings in the paper and we did not include these variables in analyses, as these were not theoretically meaningful comparisons.
Population characteristics	Other characteristics of the study samples (e.g., age, student status, employment status) are displayed in Supplementary Figures 11 and 12.
Recruitment	Participants were recruited via convenience sampling through the online panel provider Prolific. We collected nationally-representative samples in the United Kingdom and the United States, limiting any potential self-selection biases.
Ethics oversight	This research was approved by the UCL Department of Psychology Ethics Committee (project: 3720/002) and ratified by the University of Auckland Human Participants Ethics Committee.

Note that full information on the approval of the study protocol must also be provided in the manuscript.

Field-specific reporting

Please select the one below that is the best fit for your research. If you are not sure, read the appropriate sections before making your selection.

☐ Life sciences ☒ Behavioural & social sciences ☐ Ecological, evolutionary & environmental sciences

For a reference copy of the document with all sections, see nature.com/documents/nr-reporting-summary-flat.pdf

Behavioural & social sciences study design

All studies must disclose on these points even when the disclosure is negative.

Study description	The study consisted of two quantitative surveys (a survey with economic games and a follow-up survey).
Research sample	The research sample consists of Prolific participants from the United Kingdom (n = 1014) and the United States (n = 996). These samples were nationally-representative with respect to age, gender, and ethnicity. Sample characteristics are displayed in Supplementary Figures 11 and 12.
Sampling strategy	We recruited participants via convenience sampling through Prolific. We aimed to recruit the largest sample we could with available funds, allowing for potential attrition from the first survey to the second.
Data collection	We collected data using the survey platform Qualtrics.
Timing	We began data collection in the United Kingdom on 28th November 2022, with participants returning to complete the follow-up survey on 5th December 2022. We then ran a second wave of data collection in the United States on 20th June 2023, with participants returning to complete the follow-up survey on 27th June 2023.
Data exclusions	We pre-registered that we would exclude participants who failed any of the attention checks, sped through the surveys (i.e., two standard deviations below the median duration), or flatlined (i.e., provided identical responses to matrix questions). We also stated that we would exclude data for particular games if participants failed the comprehension question for that game. We followed our pre-registered plan of conducting analyses with and without these exclusions (analyses without exclusions are reported in the Supplementary Material).
Non-participation	In the United Kingdom, 46 participants dropped out (i.e., completed the economic games survey but did not return to complete the follow-up survey). In the United States, 48 participants dropped out.
Randomization	In the first survey, we presented participants with all six economic games in a randomised order, with punishment decisions randomised within games. In the follow-up survey, participants completed blocks of questions on demographics, personality, Social Value Orientation, political ideology, and religious views in a random order, with questions randomised within blocks. Randomisation was implemented with the randomizer function in the Qualtrics survey flow.

Reporting for specific materials, systems and methods

We require information from authors about some types of materials, experimental systems and methods used in many studies. Here, indicate whether each material, system or method listed is relevant to your study. If you are not sure if a list item applies to your research, read the appropriate section before selecting a response.

Materials & experimental systems

n/a	Involved in the study
☒	☐ Antibodies
☒	☐ Eukaryotic cell lines
☒	☐ Palaeontology and archaeology
☒	☐ Animals and other organisms
☒	☐ Clinical data
☒	☐ Dual use research of concern
☒	☐ Plants

Methods

n/a	Involved in the study
☒	☐ ChIP-seq
☒	☐ Flow cytometry
☒	☐ MRI-based neuroimaging

Plants

Seed stocks	*Report on the source of all seed stocks or other plant material used. If applicable, state the seed stock centre and catalogue number. If plant specimens were collected from the field, describe the collection location, date and sampling procedures.*
Novel plant genotypes	*Describe the methods by which all novel plant genotypes were produced. This includes those generated by transgenic approaches, gene editing, chemical/radiation-based mutagenesis and hybridization. For transgenic lines, describe the transformation method, the number of independent lines analyzed and the generation upon which experiments were performed. For gene-edited lines, describe the editor used, the endogenous sequence targeted for editing, the targeting guide RNA sequence (if applicable) and how the editor was applied.*
Authentication	*Describe any authentication procedures for each seed stock used or novel genotype generated. Describe any experiments used to assess the effect of a mutation and, where applicable, how potential secondary effects (e.g. second site T-DNA insertions, mosiacism, off-target gene editing) were examined.*
